# MET and FASN as Prognostic Biomarkers of Triple Negative Breast Cancer: A Systematic Evidence Landscape of Clinical Study

**DOI:** 10.3389/fonc.2021.604801

**Published:** 2021-05-27

**Authors:** Weihua Jiang, Xiao-Liang Xing, Chenguang Zhang, Lina Yi, Wenting Xu, Jianghua Ou, Ning Zhu

**Affiliations:** ^1^ The Affiliated Tumor Hospital of Xinjiang Medical University, Wulumuqi, China; ^2^ Hunan University of Medicine, Huaihua, China

**Keywords:** TNBC, MET, FASN, recurrence, metastasis, prognosis biomarker

## Abstract

**Background:**

To know the expression of Mesenchymal–Epithelial Transition factor (MET) and Fatty Acid Synthase (FASN) in Triple Negative Breast Cancer (TNBC) patients, as well as its relationship with clinical pathological characteristic and prognosis.

**Methods:**

we used immunohistochemistry staining to detect the expression of MET and FASN for those 218 TNBC patients, and analyze their relationship with the clinical pathological characteristic and prognosis.

**Results:**

130 and 65 out of 218 TNBC patients were positive for MET in the cancer and adjacent tissues respectively. 142 and 30 out of 218 TNBC patients were positive for FASN in the cancer and adjacent tissues respectively. Positive expression of MET and FASN were significantly correlated with lymph node metastasis, pathological TNM, and pathological Stage. In addition, the positive expression of MET and FASN were correlated with recurrence and metastasis. The combined use of MET and FASN can better predict the survival condition.

**Conclusions:**

Our results indicated that MET and FASN showed good predictive ability for TNBC. Combined use of MET and FASN were recommended in order to make a more accurate prognosis for TNBC.

## Introduction

Breast Cancer (BC) is the most commonly diagnosed cancer and the leading cause of cancer death (1). In 2018, there are almost 2.1 million new cases of BC have been diagnosed which account for about 1/4 cancer cases among women (1). Depend on molecular and histological evidences, BC could be classified into BC expressing hormone receptor (estrogen receptor (ER^+^) or progesterone receptor (PR^+^)), BC expressing human epidermal receptor 2 (HER2^+^) and triple-negative breast cancer (TNBC) (ER^−^, PR^−^, HER2^−^) (2, 3). About 25% of early breast cancer patients still experience local recurrence and develop distant metastases after active treatment (4). TNBC is one of the most aggressive subtypes of BC, which accounts for about 10–15% of all breast cancers (5). Currently, there are few effective treatments for TNBC. And the prognosis of TNBC is worse than that of non-TNBC (6, 7). Previous studies indicated that the treatment approaches for BC should be based on their molecular characteristics. Therefore, it is important to search for suitable prognostic biomarkers for the prognosis diagnosis and clinical treatment of TNBC.

MET (Mesenchymal–epithelial transition factor) is a receptor tyrosine kinase which could be activated by its ligand hepatocyte growth factor (HGF). The activation of MET and its downstream signaling pathway involved in a number of important biological activities, including tumor cells growth, proliferation and metastasis (8, 9). Recently, increasing evidences indicated that MET is closely correlated with the development of BC (10, 11). MET positive TNBC patients have a shorter overall survival, their death risk is 1.8 times that of negative patients (12). MET is highly expressed in TNBC cell lines. And inhibition of MET could reduce cell proliferation and migration (12). Fatty acid synthase (FASN) is a key enzyme in fat biosynthesis, which plays an important role in regulating the expression of genes involved in apoptosis and DNA repair (13). It is highly expressed in various sex hormone-related malignant tumors and closely related to the proliferation, invasion, metastasis, drug resistance, and apoptosis of tumor cells (13). Current studies have shown that FASN is closely related to the development of BC (14–16). The expression degree of FASN is positively correlated with malignant degree, recurrence, metastasis and death of tumor (17).

In this study, we detect the expression of MET and FASN in TNBC, and analysis the correlation of MET and FASN with clinic pathological characteristics. 130 and 65 out of 218 TNBC patients were positive for MET in the cancer and adjacent tissues respectively. 142 and 30 out of 218 TNBC patients were positive for FASN in the cancer and adjacent tissues respectively. Positive expression of MET and FASN were correlated with lymph node metastasis, pathological TNM, and pathological Stage. In addition, the positive expression of MET and FASN were correlated with recurrence and metastasis. The combined use of MET and FASN can better predict the survival condition. Combined analysis indicated that the TNBC patients with MET and FASN positive expression displayed a worse overall survival. And the AUC was higher than 0.6, which indicated that the combined used of MET and FASN could predict the survival situation more accurately.

## Materials and Methods

### Patients

This study was carried out in accordance with the World Medical Association’s Declaration of Helsinki and approved by the Research Ethics Committee in The Affiliated Tumor Hospital of Xinjiang Medical University. A total of 218 TNBC patients which confirmed by pathological examination in the affiliated tumor hospital of Xinjiang medical university from 2013 to 2015 were collected (**Table 1**).

**Table 1:** Clinical characteristic.

Category		Case (218)
Age	<40	65
	≥40	153
Operation method	Improved radical mastectomy	127
	Breast-conserved radical mastectomy	72
	Breast reconstruction surgery	19
Treatment	Neoadjuvant chemotherapy	72
	Adjuvant chemotherapy	146

### Immunohistochemistry

MET antibody (ZA-0636) was purchased from Beijing Zhong Shan Jin Qiao Biotechnology Company. FASN antibody (FNab-03019) was purchased from Wuhan Enfei Biotechnology Company. The operation of immunohistochemistry was strictly in accordance with the content of quality control. (1) The surgically resected tissue of TNBC patients was treated with 10% formalin fixation and paraffin embedding, and then histological sections were performed (4μm). (2) After routine dewaxing and rehydration, we washed the histopathological sections with PBS (pH =7.4) for three times at 5 min interval. After heat-induced epitope recovery, we washed the histopathological sections with ddH_2_O. (3) We used hydrogen peroxide solution to block endogenous catalase activity (at room temperature, 10 min). (4) The primary antibody (Dilution: MET, 1:200; FASN, 1:100) was incubated at 4°C overnight. Then washed it with PBS for 5 min and repeated three times. (5) Biotin conjugated secondary antibody was added and incubated at room temperature for 30 min, following with three times wash by PBS for 5 min/time. (6) Add chromogenic reagent for incubation 3 min, hematoxylin dyeing for nuclear staining and dehydration for seal the slide.

### Criteria for Result Analysis

**Figure 1:**
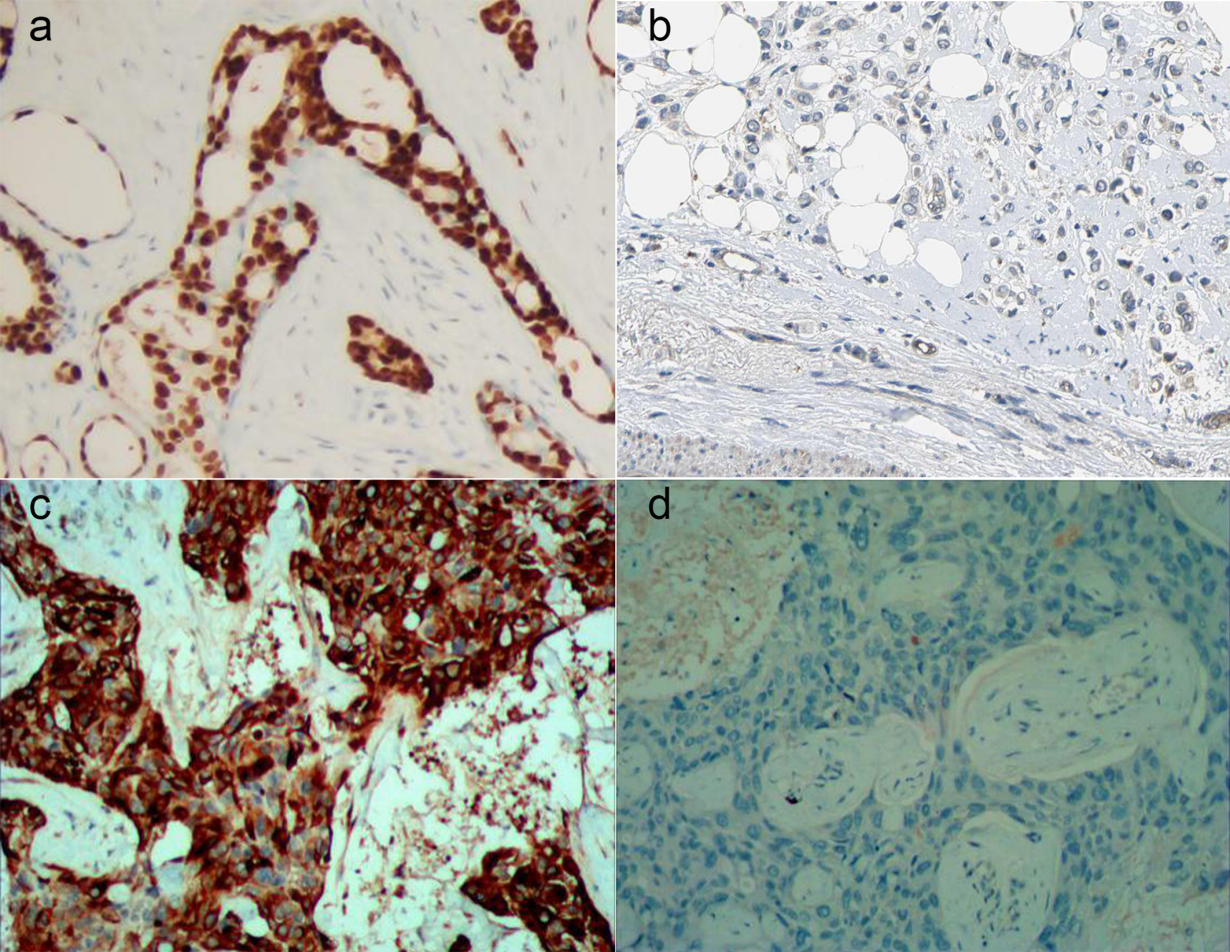
Expression detection of MET and FASN by Immunohistochemistry. **(A, B)**, positive **(A)** and negative **(B)** expression of MET by immunohistochemistry. **(C, D)**, positive **(C)** and negative **(D)** expression of FASN by immunohistochemistry.

The expression of MET and FASN protein was observed by two-person use double-blind method. All slides contain multiple tissue sections and set antibody or PBS as positive control and negative control. The results were determined by two-person double-blind observation. The ratio of positive cells and the intensity of cell staining were considered to be the judgment criteria. The score criteria for the ratio of MET positive cells were displayed as follows: negative represented 0, <25% represented 1, 26–50% represented 2, 51–75% represented 3, and >75% represented 4. The score criteria for the dyeing intensity were displayed as follows: negative represented 0, light yellow represented 1, dark yellow represented 2, and brown represented 3. A patient is considered to positive for MET if the sum of those two indexes was greater than 4, and negative if they are not (**Figures 1A, B**) (18). The score criteria for the ratio of FASN positive cells were displayed as follows: ≤1% represented 0, 2–10% represented 1, 11–50% represented 2, 51–80% represented 3, and ≥81% represented 4. The score criteria for the dyeing intensity were displayed as follows: negative represented 0, light brown represented 1, brown represented 2, and dark brown represented 3. A patient is considered to positive for MET if the sum of those two indexes was greater than 4, and negative if they are not (**Figures 1C, D**) (19).

### Follow-Up Records

The follow-up time of this study was calculated from the postoperative period. The primary survival assessment was disease-free survival (DFS) for five years. DFS time referred to the time from the first day after surgery to local recurrence, regional recurrence, or distant metastasis.

### Construction of the TNBC Prognostic Model

We first marked patients with both MET and FASN positive as 2 points, marked patients with MET or FASN positive as 1 point, marked patients with MET and FASN negative as 0 point. After the assignment, we classify the sample as following:①Group 1: 1 and 2 points, Group 2: 0 point. ②Group 1: 2 points, Group 2: 0 and 1 point. After group divided, we carried our univariate Cox hazards regression analysis and time-dependent receiver operating characteristic (ROC) curves analysis.

### Statistical Analysis

IBM SPSS 22 software was used to carry out univariate and multivariate Cox regression analysis, and chi-square test analysis, Kaplan–Meier analysis. P <0.05 was considered statistically significant.

## Results

### Expression of MET and FASN in Cancer and Adjacent Tissues of TNBC

In this present study, we used IHC staining to detect the expression of MET and FASN. We found that 130 and 65 out of 218 TNBC patients were positive for MET in the cancer and adjacent tissues respectively. The positive rates were 59.6 and 29.8% (**Table 2**). 142 and 30 out of 218 TNBC patients were positive for FASN in the cancer and adjacent tissues respectively. The positive rates were 65.1 and 13.8%. The positive rate for MET and FASN in cancer tissues of TNBC were higher significantly than those in the adjacent tissue (**Table 2**). There were 83 TNBC patients with both positive expression of MET and FASN.

**Table 2:** Expression of MET and FASN in cancer and adjacent tissues.

Group	MET		FASN	
	Positive	Negative	Positive	Negative
Cancer Tissue	130 (59.6)	88 (40.4)	142 (65.1)	76 (34.9)
Adjacent Tissue	65 (29.8)	153 (70.2)	30 (13.8)	188 (86.2)
Chi-square value	39.19		120.4	
p value	<0.001		<0.001	

### The Relationship of MET and FASN With the Clinical Pathological Features

To know the relationship of MET and FASN with the clinical pathological features, we carried out the correlation analysis and found that the positive expression of MET and FASN were significantly correlated with lymph node metastasis, pathological TNM, and pathological Stage. In addition, we also found that the positive expression of FASN was significantly correlated with diabetes and body mass index (**Table 3**). Those results indicated that MET and FASN may be the factor affecting the progression of TNBC.

**Table 3:** The relationship of MET and FASN with the clinical pathological features in TNBC.

Clinical pathological features		n	MET		chi-square value	p value	FASN		chi-square value	p value
			positive	negative			positive	negative		
Tumor size (cm)	≤2	55	29 (52.7)	26 (47.3)	1.648	0.439	30 (54.5)	25 (45.5)	4.114	0.128
	2–5	124	78 (62.9)	46 (37.1)			87 (70.2)	37 (29.8)		
	≥5	39	23 (59.0)	16 (41.0)			25 (64.1)	14 (35.9)		
Lymph node	metastasis	66	46 (69.7)	20 (30.3)	3.983	**0.046**	50 (75.8)	16 (24.2)	4.701	**0.030**
	no-metastasis	152	84 (55.3)	68 (44.7)			92 (60.5)	60 (39.5)		
Pathological TNM	I	50	22 (44.0)	28 (56.0)	6.643	**0.036**	21 (42.0)	29 (58.0)	16.30	**0.001**
	II	110	70 (63.6)	40 (36.4)			79 (71.8)	31 (28.2)		
	III	58	38 (65.5)	20 (34.5)			42 (72.4)	16 (27.6)		
Pathologic Stage	I	15	5 (33.3)	10 (66.7)	6.365	**0.041**	4 (26.7)	11 (73.3)	11.18	**0.004**
	II	129	75 (58.1)	54 (41.9)			85 (65.9)	44 (34.1)		
	III	74	50 (67.6)	24 (32.4)			53 (71.6)	21 (28.4)		
Diabetes	Positive	29	16 (55.2)	13 (44.8)	0.276	0.599	24 (82.8)	5 (17.2)	4.574	**0.032**
	Negative	189	114 (60.3)	75 (39.7)			118 (62.4)	71 (37.6)		
Body mass index	<18.5	4	1 (25.0)	3 (75.0)	2.053	0.561	0 (0.0)	4 (100)	13.33	**0.004**
	18.5–23.9	102	62 (60.8)	40 (41.2)			60 (58.8)	42 (41.2)		
	24–27.9	70	42 (60.0)	28 (40.0)			49 (70.0)	21 (30.0)		
	≥28	42	25 (59.5)	17 (40.5)			33 (78.6)	9 (21.4)		

Since the P value is less than 0.05, we highlighted it in bold.

### Associated Analysis of Recurrence and Metastasis With MET and FASN

To know the relationship of positive expression of MET and FASN with their recurrence and metastasis, we followed up those 218 patients for five years. Of 218, 54 TNBC patients had cancer recurrence and metastasis. For MET, we found the ratio of DFS in positive expression group and negative expression group were 70.0 and 83.0% respectively. The positive expression of MET was significantly correlated with cancer recurrence and metastasis (X^2^ = 4.726, p = 0.030). For FASN, we also found the ratio of DFS in positive expression group and negative expression group were 70.4 and 84.2% respectively. The positive expression of FASN was significantly correlated with cancer recurrence and metastasis (X^2^ = 5.505, p = 0.025) (**Table 4**).

**Table 4:** Correlation analysis of MET and FASN with the recurrence and metastasis.

Group	MET		FASN	
	Positive	Negative	Positive	Negative
Recurred and Metastasis (%)	39 (72.2)	15 (27.8)	42 (77.8)	12 (22.2)
No- recurred and metastasis (%)	91 (55.5)	73 (44.5)	100 (61.0)	64 (39.0)
chi-square value	4.726		5.505	
p value	0.030		0.025	

At meanwhile, we carried out univariate Cox regression analysis for MET and FASN. The TNBC patients with negative expression of MET (P = 0.036) and FASN (P = 0.029) displayed a better overall survival. The result of multivariate Cox regression analysis indicated that the expression of MET (B = 0.685, P = 0.025) and FASN (B = 0.757, P = 0.021) were still correlated with the overall survival.

**Figure 2:**
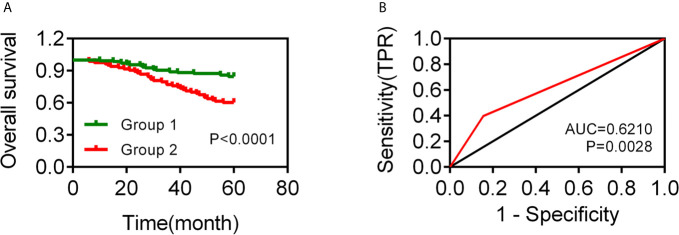
Analysis of the TNBC prognostic model based on MET and FASN expression. **(A)** The overall survival curve of TNBC patients with group 1 (2 points, both positive expression of MET and FASN) and group 2 (0 and 1 point, positive expression of MET or FASN, and negative expression of MET and FASN). **(B)** The AUC value of ROC curve in 5-year for TNBC patients.

To know whether MET and FASN could be combined for TNBC diagnostic analysis, we divided the TNBC patients into two groups as following: Group 1: 1 and 2 points, Group 2: 0 point. We carried out univariate Cox regression analysis and found that there was no significantly difference in overall survival between groups 1 and 2. And then, we divided the TNBC patients into two groups as following: Group 1: 2 points, Group 2: 0 and 1 point. We carried out univariate Cox regression analysis and found that group 1 displayed a worse overall survival (**Figure 2A**). The time-dependent receiver operating characteristic (ROC) curves have area under curve (AUC) values higher than 0.5 significantly, which were 0.6210 (p = 0.0028) (**Figure 2B**).

## Discussions

BC is the most commonly diagnosed cancer and the leading cause of cancer death (1). There are almost 2.1 million new cases of BC have been diagnosed which account for about 1/4 cancer cases among women in 2018 (1). TNBC is one of the most aggressive subtypes of BC. It is important to find suitable biomarkers in TNBC patients for prognosis diagnosis.

MET is a protein encoded by the proto-oncogene *MET* which is located in 7q21-q31 of chromosome 7. MET is involved in the activation of multiple signal transduction pathways, including RAS, PI3K and -catenin pathways, which play an important role in the occurrence and development of cancer (20–22). Previous studies have demonstrated that abnormal activation of MET signaling pathway could promote neovascularization, lymphangiogenesis, proliferation and differentiation of tumor cells, malignant tumor invasion and metastasis (23). For example, Bleau et al. (24) found that miR-146a targets c-met and abolishes colorectal cancer liver metastasis. Guo et al. (25) found miRNA-454-3p inhibits cervical cancer cell invasion and migration by targeting Met. Han et al. (26) found miR-1 could inhibit gastric cancer cell proliferation and migration by targeting MET. Lee et al. (27) found that inhibition of Met and VEGFR2 in osteoblasts reduced RANKL and M-CSF expression, and associated with reduction of tumor-induced osteolysis. In addition, previous studies indicated abnormal activation of MET was also involved in the development of BC. For example, Meng et al. (11) found that EGFL9 could promote breast cancer metastasis by inducing MET activation and metabolic reprogramming. Zeng et al. (10) found that FEN1 mediates miR-200a methylation and promotes BC cell growth *via* MET and EGFR signaling pathway. Those reports indicated MET played important role in the development of cancer, including BC. In this present study, we found 130 out of 218 TNBC patients (59.6%) was positive for MET in the cancer tissue which were significantly higher than that in adjacent tissues. We also found the positive expression of MET was associated with lymph node metastasis, pathological TNM, and pathological stage in TNBC patients significantly. Our results reinforced the relationship of MET with the development of cancers. In addition, we found that the TNBC patients with positive expression of MET exhibited poorer overall survival. Our results not only enhanced the relationship between MET expression and BC survival rate, but also suggested that MET expression was also closely related to the overall survival of BC subtype TNBC (12). Our results indicated that MET could be a prognostic biomarker for TNBC which was similar to previous studies (28, 29).

FASN is an enzyme that encoded by the *FASN* gene, could catalyzes fatty acid synthesis. Previous study indicated that *FASN* could be a possible oncogene (30). Orlistat was a gastrointestinal lipase inhibitor which could be a potential medicine for cancers (31). Inhibition of FASN could suppress the proliferation, invasion, and metastasis of cancer cells (15, 32, 33). In the present study, we found that 142 out of 218 TNBC patients (65.1%) were positive for FASN in cancer tissue which was significantly higher than that in adjacent tissues. Positive expression of FASN was associated with lymph node metastasis, TNM stage, histological grading, diabetes, and body mass index. In addition, we also found FASN was correlated with the overall survival of TNBC patients. TNBC patients with positive expression of FASN exhibited poorer overall survival. These results indicated that FASN could be a prognostic biomarker for TNBC patients. Our result was consistent with previous reports showed that FASN was an indicator of poor prognosis (34).

## Conclusions

Combined analysis indicated that the TNBC patients with MET and FASN positive expression displayed a worse overall survival. And the AUC was higher than 0.6, which indicated that the combined used of MET and FASN could predict the survival situation more accurately.

## Data Availability Statement

The raw data supporting the conclusions of this article will be made available by the authors, without undue reservation.

## Ethics Statement

The studies involving human participants were reviewed and approved by Research Ethics Committee in The Affiliated Tumor Hospital of Xinjiang Medical University. The patients/participants provided their written informed consent to participate in this study.

## Author Contributions

WJ and NZ conceived and designed the experiments. CZ, LY, and WX performed the experiments. JO, and X-LX helped to analyze the data. X-LX wrote the paper. All authors contributed to the article and approved the submitted version.

## Funding

This project is financially supported by the Natural Science Foundation of Xinjiang (2017D01C407).

## Conflict of Interest

The authors declare that the research was conducted in the absence of any commercial or financial relationships that could be construed as a potential conflict of interest.
